# Wildlife Pathogens and Zoonotic Disease Risk Assessment in Vietnam: A Wildlife Trade Hotspot

**DOI:** 10.1155/tbed/4926262

**Published:** 2025-06-19

**Authors:** Alice Latinne, Pawin Padungtod

**Affiliations:** ^1^Emergency Centre for Transboundary Animal Diseases (ECTAD), Food and Agriculture Organization of the United Nations (FAO) Representation in Viet Nam, Hanoi, Vietnam; ^2^Southeast Asia-Pacific Program, Wildlife Conservation Society, Suva, Fiji

## Abstract

Vietnam is a wildlife trade hotspot presenting multiple high-risk interfaces for pathogen spillover from wildlife to humans. However, the zoonotic disease risk remains poorly characterized in the country and needs to be assessed to better inform policy dialog and legislative reforms. A literature review was conducted to create a pathogen inventory of terrestrial vertebrates in Vietnam. Additionally, data from an existing global database were used to estimate the number of zoonotic pathogens found in different families. The literature review yielded 87 eligible records. A total of 162 pathogen species, including 22 parasites, 48 bacteria, two fungi, eight protozoans, and 82 viruses, were recorded in Vietnam in 46 families of terrestrial vertebrates belonging to four classes and 18 orders. The highest number of pathogens was observed in Muridae (rats and mice), followed by Pythonidae (pythons) and Cercopithecidae (Old World monkeys). A total of 12 out of 29 priority zoonoses in Vietnam were reported in 27 terrestrial wildlife host families. Zoonotic pathogens were reported at 11 human–wildlife interfaces. Most detections of priority zoonotic pathogens were made in free-ranging animals as well as in wildlife farms and primate facilities. A risk assessment, based on the number of zoonotic pathogens found, suggested that facilities with a very high risk of zoonotic spillover include bushmeat markets in cities and town, wildlife farms, restaurants and rescue centers engaged in trading, housing and breeding birds belonging to the Columbidae, Phasianidae, Ardeidae families, and mammals belonging to the Cervidae, Suidae, Felidae, Ursidae, Mustelidae, Cercopithecidae, Muridae, and Sciuridae families. These supply chain nodes where wildlife families are in contact with human populations should be strictly regulated and monitored, with stricter biosecurity measures. Breeding of several species belonging to high-risk and medium-risk wildlife families together in the same captive facility should be banned to reduce the risk of pathogen-host jumps.

## 1. Introduction

The outbreaks and pandemics resulting from the emergence of novel zoonotic viruses can significantly harm human societies and economic growth worldwide [1, 2]. Since 1950, the global annual loss in world gross national income from viral zoonotic disease outbreaks is estimated at $212 billion [2]. The global COVID-19 pandemic that followed the emergence of severe acute respiratory syndrome (SARS)-CoV-2 in China in 2019 disrupted livelihoods worldwide and resulted in over 775 million confirmed cases and over 7 million deaths globally as of April 2024 [3]. Economic computations showed that prevention of zoonotic pandemics through earlier detection of pathogen spillover, improved management of wildlife trade, and a decrease in deforestation would cost much less than the costs of pandemics [1, 2].

Wildlife supply chains allow close contact between humans and wild animals from various origins and present a high risk for the emergence and transmission of zoonotic pathogens that could potentially cause future pandemics, as evidenced by the emergence of the Ebola virus in West Africa and SARS-CoV-1, SARS-CoV-2, Middle East respiratory syndrome (MERS)-CoV, and influenza A subtypes [4–6]. New viruses created through the exchange of viral components among wildlife species moving through wildlife supply chains may cause infections in humans and lead to outbreaks [7, 8].

The wildlife supply chains and conditions bringing humans in close contact with wildlife are common in Vietnam and multiple high-risk interfaces for pathogen spillover from wildlife to humans have been identified in the country [9]. Commercial wildlife farming has rapidly expanded in Vietnam over the last decades, with 7184 registered farms in 2021 and 454 species of captive wild animals identified in the period 2015–2021 [10]. However, a decrease in the number of facilities has been observed in recent years [10, 11]. In 2021, the four most common species raised in Vietnamese wildlife farms were the freshwater crocodile (*Crocodylus siamensis* Schneider, 1801), the Asian palm civet (*Paradoxurus hermaphroditus* Pallas, 1777), the porcupine (*Hystrix brachyura* Linnaeus, 1758), and the sambar deer (*Cervus unicolor* Kerr, 1792) [10]. Lack of biosecurity measures and high-risk practices for zoonotic spillovers, such as mixing of multiple taxa in the same facility, frequent restocking using wild-caught animals, lack of quarantine facility, poor hygiene, and sales of sick individuals for human consumption, were reported in those facilities [12–14]. The lack of a systematic surveillance system to monitor wild animal health in captive wildlife facilities also increases the risk of zoonotic disease outbreaks along the wildlife supply chains in Vietnam [15].

The rat trade that expands over the entire Mekong Delta region in Vietnam and Cambodia also represents an important zoonotic risk [16, 17]. In the early 2000s, the annual quantity of rat meat traded for human consumption in the region was 3300–3600 tons of live rats [18]. Vietnam is also a key transit area for transporting illegal wildlife animals and products to other countries in Asia, mostly China [19, 20]. Both legal and illegal international wildlife trade pose a high risk for zoonotic pathogen transmission and disease emergence as they bring a diversity of wildlife species and humans in close contact [5]. Activities related to hunting, trapping, butchering, transporting, sale, and consumption of wild animals may result in the introduction of zoonotic and exotic pathogens in regions where they were absent, in pathogen transmissions to new hosts, and in the amplification of pathogens [4, 5].

Despite the presence of those high-risk interfaces, the zoonotic disease risk remains poorly characterized and assessed in Vietnam. A recent survey identified 29 priority zoonotic pathogens and diseases commonly detected in Vietnam that would require better detection and prevention using a One Health approach [21]. However, wildlife hosts of these pathogens and interfaces at which they spill over into humans remain mostly unknown. Filling these data gaps is critical to better inform policy and legislative reforms in Vietnam [15].

This review paper aims to assess zoonotic pathogen risk in wildlife in Vietnam and identify high-risk interfaces to better detect and prevent zoonotic disease emergence. More specifically, our objectives were to (i) compile data available in the English literature and create an inventory of pathogens and parasites identified in wildlife species in Vietnam in order to provide an accurate overview of the zoonotic pathogen diversity in wildlife in Vietnam and identify research gaps and priorities for future research; (ii) map those pathogens and parasites on the wildlife value chain in Vietnam and identify the interfaces at which the priority zoonotic diseases have been detected; and (iii) identify host species and value chain nodes presenting a high zoonotic risk using a semiquantitative risk assessment framework.

## 2. Materials and Methods

### 2.1. Literature Review

The main literature review was conducted using the electronic databases PubMed and Google Scholar and the following search terms:

i. bacteria^*∗*^ OR virus^*∗*^ OR parasite^*∗*^ OR fung^*∗*^ OR helminth^*∗*^ OR pathogen^*∗*^ OR viral^*∗*^ OR zoono^*∗*^
ii. AND: animal OR wildlife OR “wild life” OR “wild-life” OR “wild animal” OR “wild bird” OR pet^*∗*^ OR park OR zoo^*∗*^
iii. AND: Vietnam OR “Viet nam” OR viet^*∗*^

Additional searches were conducted by replacing the search terms in (i) with the 29 priority zoonoses identified in Vietnam [21] and viral families with high zoonotic potential (filo^*∗*^ OR rhabdo^*∗*^ OR corona^*∗*^ OR flavi^*∗*^ OR paramyxo^*∗*^).

**Figure 1:**
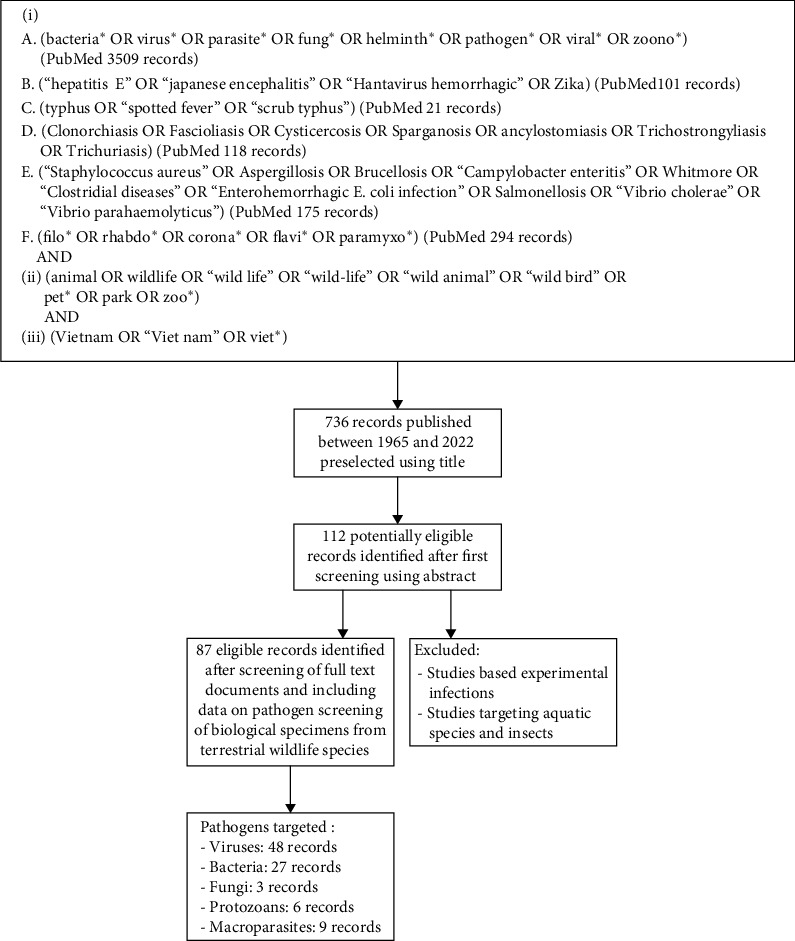
Schematic flow chart of the literature selection for the review.

A first assessment of all records published between 1965 and August 2022 was conducted using their title and abstract (Figure 1). Selected publications were then screened using full-text documents. Records were eligible for data extraction if they were published in English and included data on pathogen screening of biological specimens collected from terrestrial wildlife species in Vietnam or exported from Vietnam. All terrestrial vertebrate species belonging to the animal classes Amphibia (amphibians), Reptilia (reptiles), Aves (birds) and Mammalia (mammals) and not commonly raised as pets (dogs and cats), livestock (cows, buffalos, swine, and goats) or poultry (chickens and ducks) in Vietnam were considered as wildlife species. Studies based on experimental infections in laboratory conditions or targeting aquatic species and insects were excluded (Figure 1). Studies based on pathogen detection from vectors (ticks, mites, and fleas, etc.) were included only if the animal host of the vector was unambiguously identified in the study.

### 2.2. Data Extraction From Eligible Records

Data were extracted from eligible publications using a standardized spreadsheet to create a pathogen inventory of terrestrial vertebrates in Vietnam (Supporting Information S1). The following data items were recorded for each publication: host genus, species and common name, type of pathogens targeted by the study, species name of the pathogen detected, prevalence, detection method, country, locality, year, and the type of human–wildlife interface at which the pathogen was detected.

After extraction from all publications, data were cleaned, and host and pathogen/parasite names were standardized using the NCBI taxonomic database. Host and pathogen taxonomy information were added to the spreadsheet. The latest recommendations from the International Committee on Taxonomy of Viruses (ICTVs) were used for virus names. All pathogens/parasites were classified as zoonotic (human infections recorded) or not based on literature data [22, 23]. Bacterial pathogens identified at the genus level only were considered zoonotic if their genus was known to include zoonotic species.

Interface names were also standardized and 13 types of human–wildlife interfaces were retained: wildlife farm, free-ranging animal in a farm, primate facility, restaurant, market, free-ranging animal in market, wildlife rescue center and sanctuary, zoo and recreational park, bat guano farm and guano collection, free-ranging animal in natural habitat (forests and fields), free-ranging animal in human settlements, confiscation from illegal trade, and international import. Definitions of all types of interfaces are available in Supporting Information Table S1.

### 2.3. Global Database of Host–Pathogen Associations

Data on bacterial and viral pathogens known to infect each host family were extracted from a global host–pathogen association database [23]. Pet (dogs and cats), cattle (cows, buffalos, swine, and goats), or poultry (chickens and ducks) species were removed from the dataset, and the numbers of pathogens were summarized for each host family. Zoonotic status of each pathogen was obtained from the same source [23].

### 2.4. Zoonotic Disease Risk Assessment

Due to the scarcity of data available for several host families in Vietnam that are commonly traded in the country (see Results), global data on host–pathogen associations from Shaw et al. [23] were used for this risk assessment. Our risk assessment is based on two types of risk estimates: a quantitative “taxa zoonotic risk” and a qualitative “trade point risk”. The “taxa zoonotic risk” was assigned to each wildlife taxa based on the number of zoonotic pathogens (“zoonotic risk”, high risk ≥ 50 known pathogens and ≥40 zoonotic pathogens; medium risk = 20–49 known pathogens and/or 15–39 zoonotic pathogens; low risk ≤ 19 known pathogens and ≤14 zoonotic pathogens) and the number of Vietnam priority zoonotic pathogens (“priority zoonotic risk”, high risk = ≥ 5 Vietnam priority zoonotic pathogens; medium risk = four–two Vietnam priority zoonotic pathogens; low risk = one or no Vietnam priority zoonotic pathogen) reported globally in each wildlife family. We selected the highest risk category between the zoonotic risk and the Vietnam priority zoonotic risk as the final “traded taxa risk” for each family.

For the “trade point risk”, we used an approach similar to the zoonotic disease risk assessment for wildlife trade chains and points of sale suggested by Wikramanayake et al. [24] for viral pathogens. This risk score was assessed qualitatively using three variables (transmission risk, spread potential, and zoonotic virus risk) based on market size, crowding of wildlife that creates stressful situations, hygiene conditions, number and turnover of people through the market, distance buyers may travel after visiting a market, and points along market trade chains that could allow pathogens to accumulate and amplify the potential for zoonoses [24]. As these trade conditions may also favor the spread of other types of zoonotic pathogens, such as bacteria, and are not restricted to viruses, we used the same risk scores as Wikramanayake et al. [24] in this study. Indeed, while viruses and bacteria differ in their biology and how they survive in the environment, they are transmitted in multiple ways and share several common transmission routes, such as direct contact, airborne transmission, fecal–oral routes, vector-borne transmission and indirect transmission via contaminated surfaces, that will be similarly impacted by the three variables described above. Fungi can also be transmitted through airborne spores or droplets, contact with contaminated surfaces, and fecal–oral routes. We also added three additional types of interfaces (bat guano farms, rescue centers and sanctuaries, and zoos and recreational parks) common in Vietnam and that were not included in the initial assessment of Wikramanayake et al. [24] (Supporting Information S2). Primate facilities were included within wildlife farms. Taxa zoonotic risk and trade point risk assessments were then combined in a risk matrix of traded taxa and trade points. We also assessed the cumulative zoonotic risk of raising taxa from different families in the same facility by combining the zoonotic risk of each taxa (very high risk = two high-risk taxa in the same facility; high risk = two medium-risk taxa OR one medium-risk taxa + one high-risk taxa OR one low-risk taxa + one high-risk taxa in the same facility; medium risk = two low-risk taxa OR one medium risk taxa + one low-risk taxa in the same facility).

## 3. Results

### 3.1. Literature Review Results

The first screening of records obtained from the main and additional literature searches using title and abstract identified 112 potentially eligible records (Figure 1). Screening using full-text documents yielded 87 eligible records (Figure 1), including 86 peer-reviewed publications and one ONG report, from which data were extracted (Supporting Information S3). Data generated by the USAID PREDICT project in Vietnam and publicly available in the USAID Development Data Library [25] was also included in the inventory (Supporting Information S1).

Among all eligible records, 48 targeted viruses, 27 targeted bacteria, three targeted fungi, six targeted protozoans, and nine targeted macroparasites. Animal hosts targeted by those publications belong to the classes Amphibia (four publications), Reptilia (nine publications), Aves (nine publications), and Mammals (71 publications). Among mammals, Rodentia (34 publications) and Chiroptera (20 publications) were the most studied orders (Supporting Information Table S2).

### 3.2. Pathogens Associated With Terrestrial Vertebrates in Vietnam

A total of 162 pathogens/parasite species, including 22 parasites, 48 bacteria, two fungi, eight protozoans, and 82 viruses, were recorded in Vietnam in 46 families of terrestrial wildlife vertebrates belonging to four classes and 18 orders. Most of these pathogens, including a large proportion of viruses (81/82), were reported in Mammalia species (Table 1 and Supporting Information Table S3). It is important to note that families of some wildlife species commonly raised in wildlife farms in Vietnam, such as the Oriental rat snake (Colubridae), Siamese crocodile (Crocodylidae), sambar deer (Cervidae), and sika deer (Cervidae) [10] were not targeted by any of the publications included in the literature review and were therefore not represented in our pathogen inventory of terrestrial wildlife vertebrates in Vietnam (Table 1).

**Figure 2:**
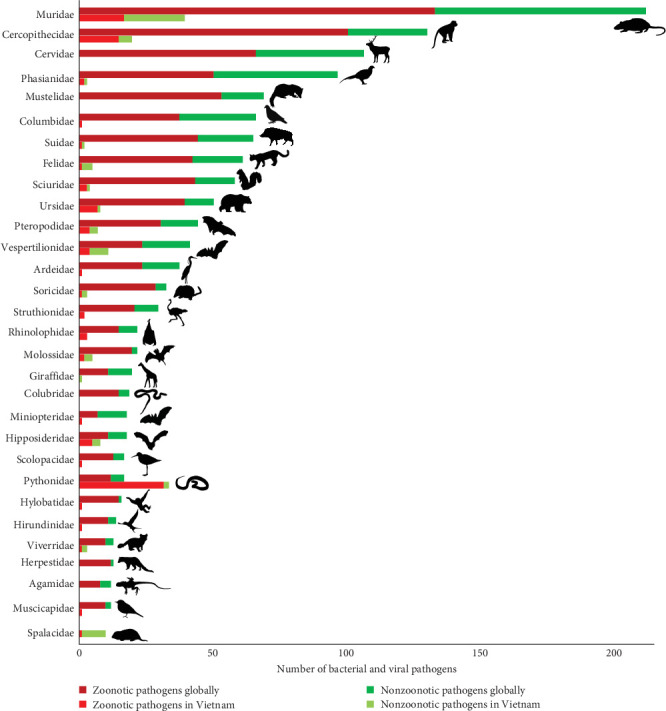
Number of zoonotic and nonzoonotic bacteria and viruses reported globally (database from Shaw et al. [23]) and in Vietnam (pathogen inventory from the literature review) in each wildlife host family. Host families presented in the figure were those included in the Vietnam pathogen inventory and for which at least 10 pathogen species were recorded. Three additional families (Cervidae, Colubridae, and Crocodylidae) known to be commonly raised in wildlife farms in Vietnam [10] were added to the figure.

The data aggregated by host families show that the highest number of pathogens, both globally and in Vietnam, was observed in Muridae (rats and mice), followed by Cercopithecidae (Old World monkeys) (Table 1 and Figure 2). A large proportion (>50%) of the pathogens hosted by these two mammal families are zoonotic. However, only a small proportion of globally reported pathogens were detected in Vietnamese wildlife (Table 1 and Figure 2). Several bird families (Phasianidae, Columbidae, and Ardeidae) and mammal families (Suidae, Felidae, Sciuridae, Ursidae, Mustelidae, and Herpestidae) host many bacteria and viruses globally, but only a few of these pathogens were reported in Vietnam (Table 1 and Figure 2). Cervidae (deers) also host many important zoonotic and nonzoonotic bacteria and viruses globally, but no pathogen was reported in this host family in Vietnam (Table 1 and Figure 2).

### 3.3. Wildlife Hosts of Priority Bacterial and Viral Zoonotic Diseases in Vietnam

**Figure 3:**
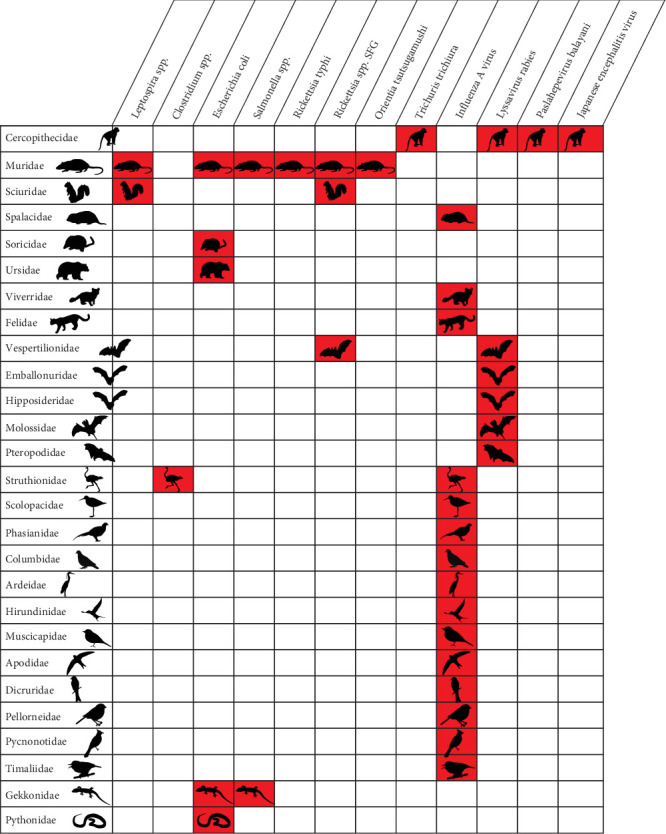
Wildlife host families of 12 priority zoonotic pathogens in Vietnam. The remaining 17 Vietnam priority zoonotic pathogens that are not included in the figure have not been identified in terrestrial vertebrate wildlife in Vietnam.

**Figure 4:**
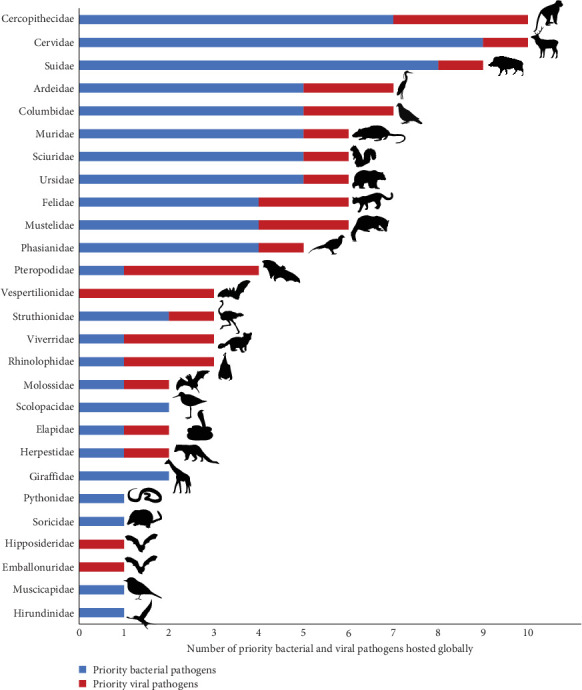
Number of Vietnam priority zoonotic pathogens (bacteria and viruses) hosted by each wildlife host family globally (data from Shaw et al. [23]).

According to our literature review, 12 of the 29 Vietnam priority zoonoses identified by Pham-Thanh et al. [21], including one helminth, four viral, and seven bacterial diseases, were reported in Vietnam in 27 terrestrial wildlife host families (Figure 3). The Influenza A virus was reported in all bird families included in the inventory as well as in several mammal families, including Spalacidae (bamboo rats), Viverridae (civets), and Felidae (cats) (Figure 4). The Lyssavirus rabies was detected in several bat families as well as in Cercopithecidae monkeys. *Paslahepevirus balayani* (hepatitis E virus), Japanese encephalitis virus, and the helminth *Trichuris trichiura* were reported in Cercopithecidae monkeys only. The bacterial priority pathogens, including *Leptospira* spp., *Clostridium* sp., *Escherichia coli*, *Salmonella* spp., *Rickettsia typhi*, *Rickettsia* sp., and *Orientia tsutsugamushi*, were detected in Muridae (rats and mice), Sciuridae (squirrels), Soricidae (shrews), Ursidae (bears), Vespertilionidae (microbats), Struthionidae (ostriches), Gekkonidae (common geckos), and Pythonidae (pythons) (Figure 3).

According to our literature review, the remaining 17 priority zoonoses were not reported in any terrestrial wildlife species in Vietnam. However, according to global data [23], hosts of these 29 Vietnam priority zoonoses include 27 of the terrestrial vertebrate families included in our pathogen inventory (Figure 3). The highest numbers of priority bacterial and viral zoonotic pathogens are observed in Cercopithecidae (*n* = 10), Cervidae (*n* = 10), Suidae (*n* = 9), Ardeidae (*n* = 7), Columbidae (*n* = 7), Muridae (*n* = 6), Sciuridae (*n* = 6), Ursidae (*n* = 6), Felidae (*n* = 6), and Mustelidae (*n* = 6) (Figure 4).

### 3.4. Pathogens Identified at Interfaces Along the Wildlife Value Chain in Vietnam

**Figure 5:**
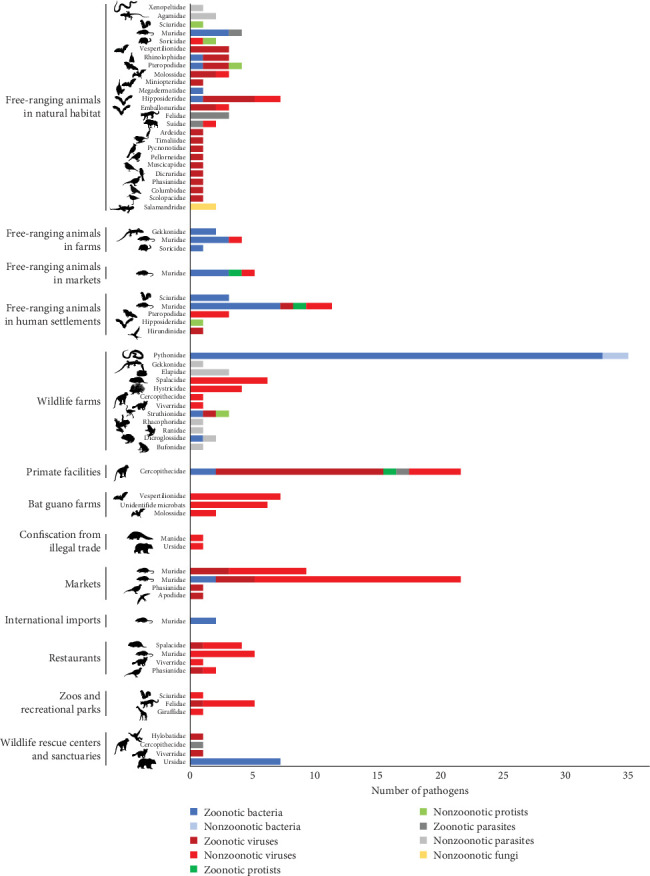
Number of pathogens detected per wildlife host families at each human–wildlife interface along the wildlife value chain in Vietnam.

**Figure 6:**
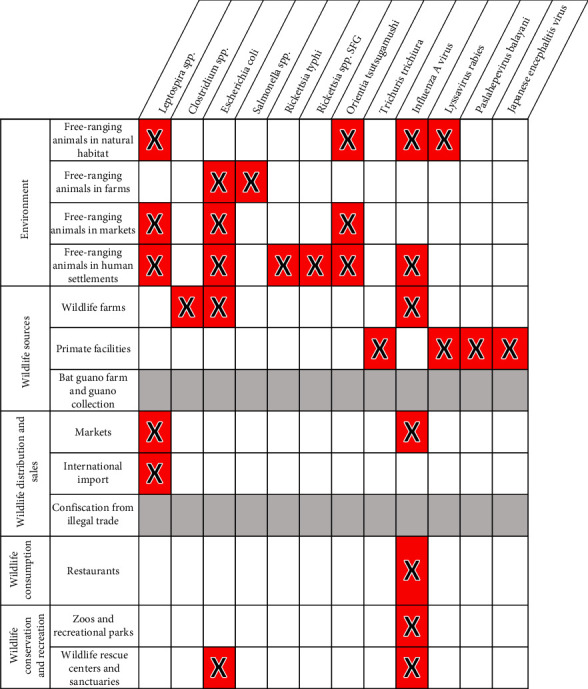
Interfaces where 12 priority zoonotic pathogens were reported in wildlife in Vietnam. The remaining 17 priority zoonotic pathogens that are not included in the figure have not been identified in terrestrial vertebrate wildlife in Vietnam.

The 13 interfaces at which pathogens and parasites were detected in Vietnam were classified under five main categories: (i) environment (free-ranging animals in natural habitat, in farms, in markets, and in human settlements), (ii) wildlife sources (wildlife farms, primate facilities and bat guano farms, and guano collection), (iii) wildlife distribution and sales (markets, confiscations from the illegal trade, and international import), (iv) wildlife consumption (restaurants), and (v) wildlife conservation and recreation (zoos and recreational parks and rescue centers and sanctuaries) (Supporting Information Table S4). Zoonotic pathogens were reported at all these interfaces in Vietnam, except in bat guano farms and in animals confiscated from the illegal trade, where the viruses detected in wildlife are not known to be able to infect humans (Figure 5 and Supporting Information Table S4). High proportions of zoonotic pathogens were observed in Cercopithecidae (Old World monkeys) and Pythonidae (pythons) raised in wildlife farms (Figure 5). Free-ranging animals sampled in animal farms, as well as in markets and human settlements, were also infected by numerous zoonotic bacteria. Most detections of priority zoonotic diseases in Vietnam were made in free-ranging animals (environment) as well as in wildlife farms and primate facilities (Figure 6).

Evidence of viral cross-taxa transmission at different interfaces was also reported in Vietnam (Supporting Information Table S5). Avian and bat coronaviruses were detected in three rodent families (Muridae, Spalacidae, and Hystricidae) in captivity or free-ranging in wildlife farms. Another bird coronavirus (duck-dominant coronavirus) was detected in Muridae rodents sold in markets, and a rhabdovirus was detected in both Cercopithecidae (Old World monkeys) and Hystricidae (porcupines) in wildlife farms. These examples confirm the risk of viral cross-taxa spillover in these facilities where different taxa are kept in close contact.

### 3.5. Zoonotic Disease Risk Assessment Along the Wildlife Supply Chain in Vietnam

High-risk host families include three bird families, Columbidae (pigeons and doves), Phasianidae (jungle fowl, peacock, pheasant, and quail), Ardeidae (wading birds), and eight mammal families, Cervidae (deers), Suidae (pigs), Felidae (cats), Ursidae (bears), Mustelidae (small carnivores), Cercopithecidae (Old World monkeys), Muridae (rats and mice), and Sciuridae (squirrels) (Supporting Information Figure S1). Medium-risk host families include two bird families, nine mammal families, and two reptile families, while all amphibian families are considered low-risk (Supporting Information Figure S1).

**Figure 7:**
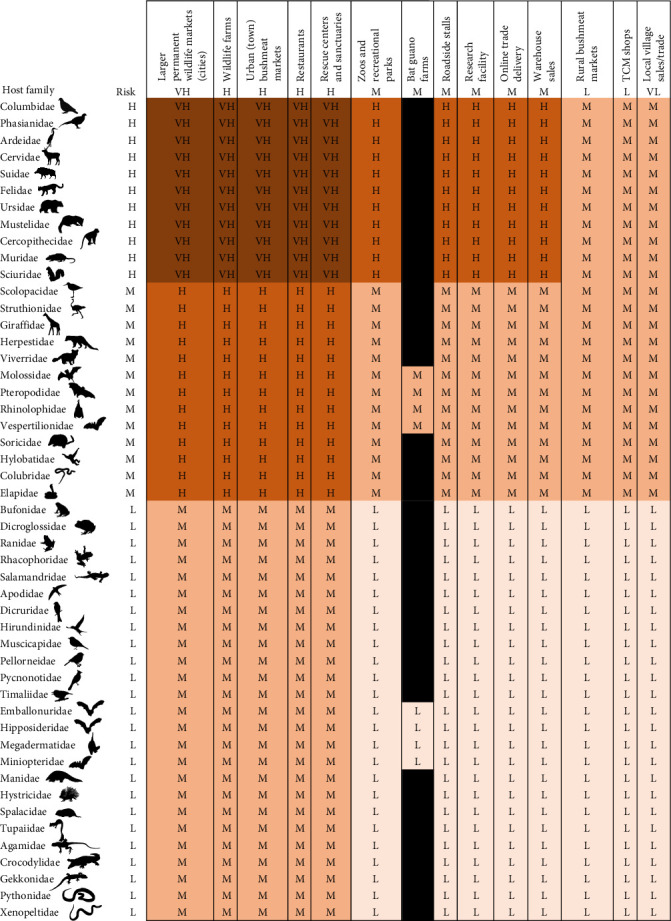
Zoonotic disease risk assessment of traded wildlife taxa at various facilities and point of sales along the wildlife supply chain (very high risk = VH, high risk = H, medium risk = M, and low risk = L). TCM, Traditional Chinese Medicine.

Facilities characterized by a very high risk of zoonotic spillover include bushmeat markets in cities and town, wildlife farms, restaurants, and rescue centers trading, housing and breeding birds belonging to Columbidae (pigeons and doves), Phasianidae (jungle fowl, peacock, pheasant, quail), Ardeidae (wading birds), and mammals belonging to Cervidae (deers), Suidae (pigs), Felidae (cats), Ursidae (bears), Mustelidae (small carnivores), Cercopithecidae (Old World monkeys), Muridae (rats and mice), and Sciuridae (squirrels) (Figure 7). Zoos, roadside stalls, research facilities, e-commerce and warehouse sales trading, and housing and breeding those animals are considered high-risk (Figure 7). Trading and breeding Scolopacidae (sandpipers), Struthionidae (ostriches), Giraffidae (giraffes), Herpestidae (mongooses), Viverridae (civets), Molossidae (free-tailed bats), Pteropodidae (fruit bats), Rhinolophidae (Horseshoe bats), Vespertilionidae (microbats), Soricidae (shrews), Hylobatidae (gibbons), Colubridae (snakes), and Elapidae (venomous snakes) in bushmeat markets in cities and town, wildlife farms, restaurants, and rescue centers is considered high risk (Figure 7).

**Figure 8:**
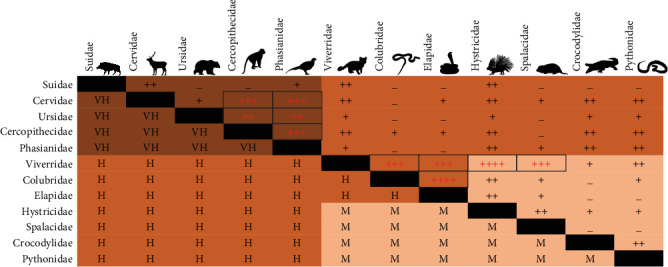
Cumulative traded taxa risk (very high risk = VH, high risk = H, and medium risk = M) in wildlife farms (below the diagonal) and number of farms breeding each combination of wildlife families in Vietnam (<10 facilities = “–”, 10–24 facilities = “+”, 25–49 facilities = “++”, 50–99 facilities = “+++”, and >100 facilities = “++++”) (above the diagonal).

We also assessed the cumulative zoonotic risk of raising taxa from different families in the same facility for 12 mammalian, avian, and reptilian families commonly raised in wildlife farms in Vietnam [10] (Figure 8). The zoonotic risk of breeding two wildlife species belonging to Suidae (pigs), Cervidae (deers), Ursidae (bears), Cercopithecidae (Old World monkeys), and Phasianidae (jungle fowl, peacock, pheasant, and quail), together in this type of facility is very high (Figure 8, below diagonal). The high numbers of facilities breeding together Cervidae or Ursidae + Cercopithecidae, Cervidae or Ursidae + Phasianidae, and Cercopithecidae + Phasianidae in Vietnam [10] indicate that these facilities represent a real zoonotic disease risk for human populations (Figure 8, above diagonal). The very high numbers of wildlife farms breeding Hystricidae + Viverridae, Elapidae + Colubridae, Colubridae + Viverridae, Elapidae + Viverridae, or Spalacidae + Viverridae also represent an important zoonotic disease risk (Figure 8, above diagonal).

## 4. Discussion

A total of 162 pathogens and parasites were reported in 46 families of terrestrial vertebrates at 13 human–wildlife interfaces in Vietnam. Rats and mice (Muridae), Old World monkeys (Cercopithecidae), and pythons (Pythonidae) are the wildlife families in which the highest numbers of pathogens have been reported in Vietnam. Our risk assessment framework based on global host–pathogen data suggested that most of the zoonotic disease risk in Vietnam is linked to eight high-risk mammal families, including Cervidae (deers), Suidae (pigs), Felidae (cats), Ursidae (bears), Mustelidae (small carnivores), Cercopithecidae (Old World monkeys), Muridae (rats and mice), Sciuridae (squirrels), and three high-risk bird families, including Columbidae (pigeons and doves), Phasianidae (jungle fowl, peacock, pheasant, and quail), and Ardeidae (wading birds), raised and/or sold in bushmeat markets in cities and town, wildlife farms, restaurants, and rescue centers.

Despite using data from a global database, our risk assessment is still likely impacted by research bias and skewed towards the most studied taxa. Additional research is urgently needed to assess the zoonotic risk associated with poorly studied key taxa along Vietnam's wildlife supply chain, such as pangolins, porcupines, civets, and bamboo rats. Wildlife families suspected to play a critical role in the transmission of zoonotic pathogens along the wildlife supply chain in Asia, such as pangolins (Manidae), small carnivores (Mustelidae), and civets (Viverridae) have been targeted by a very small number of studies in Vietnam [7, 26, 27]. Stronger scientific evidence is still needed to confirm their potential role in the emergence of zoonotic infectious diseases [28–31]. It is critical to increase our knowledge of the zoonotic risk associated with these mammal families as they are raised in wildlife farms or illegally trafficked in high numbers in Vietnam [12, 13, 26]. Future research in Vietnam should aim at increasing the diversity of wildlife hosts screened for pathogens, and the high-risk mammal and bird families mentioned above should be targeted as a priority.

The scarcity of data for many host families commonly traded in Vietnam and disparities among research protocols prevented any quantitative and statistical comparison among interfaces and wildlife supply chain nodes using our pathogen inventory to detect a potential “interface effect” on the zoonotic risk in Vietnam. Very few studies compared pathogen prevalence and diversity at different interfaces along the whole wildlife supply chain in Vietnam. Huong Nguyen Quynh et al. [7] screened rodent samples collected at different interfaces and found that the prevalence and diversity of coronaviruses in field rats increased along the wildlife supply chains from rat traders (21% rats infected) to markets (32% rats infected) and to restaurants (56% rats infected). Another study surveyed antimicrobial resistance (AMR) in *E. coli* isolates in wild mammals (rats and shrews) in Vietnam and observed that AMR was around eight times higher among isolates from wild mammals trapped in animal farms than among those trapped in natural habitat (forest and rice fields) [32]. Those studies highlight the importance of comparing data collected using standardized protocols at several human–wildlife interfaces to better characterize the role of wildlife trade and captive breeding in pathogen amplification and diversification. Future studies should simultaneously target several taxa and several nodes along the wildlife supply chain instead of focusing on a single taxon and/or single interface as it is commonly done [33–36].

Research on the zoonotic disease risk associated with the wildlife trade in other Asian countries was mostly conducted in China and Thailand, but largely remained limited to specific human–wildlife interfaces and did not target the whole supply chain. In those two countries, numerous studies revealed the large diversity of pathogens carried by captive wildlife in farms [31, 37–45] and zoos [46–50] or illegally trafficked wildlife [51–53]. Evidence of numerous spillovers of zoonotic pathogens among distantly related wildlife taxa was obtained in China [31]. Other studies highlighted the modifications in wildlife virome and gut microbiota induced by captivity [54–57].

Following the emergence of SARS-CoV-2 and the subsequent COVID-19 pandemic, China initiated legislative reforms designed to phase out the farming, trade, and consumption of terrestrial wildlife as food [58, 59]. However, neighboring countries did not follow the lead, despite calls for better regulations and wildlife trade reforms by the scientific community and various agencies [4, 60]. The lack of a large-scale disease monitoring system and limited knowledge of wildlife pathogens along the supply chain still hampers zoonotic risk assessment and the development of legislative reforms in many countries [59, 61, 62]. This risk assessment provides a basis to guide policy development in Vietnam and identify priority taxa and value chain nodes for stricter regulations and law enforcement. Very high-risk and high-risk captive facilities and supply chain nodes (i.e., wildlife and bushmeat markets, wildlife farms, restaurants, and rescue centers) where high-risk wildlife families (Figure 7) are raised and traded should be strictly regulated and controlled, with stricter biosecurity measures. Enhanced pathogen surveillance in both animal and human populations should be implemented at these facilities and target Vietnam's priority zoonotic pathogens. Breeding of several species belonging to high-risk and medium-risk wildlife families (Figure 7) together in the same captive facility should also be banned to reduce the risk of host jumps.

## 5. Conclusions

Improved pathogen surveillance and research on pathogen diversification and amplification along the wildlife supply chain are urgently needed to fill existing data gaps, as highlighted by this study and others [21, 61, 62], to better inform policy dialog and wildlife trade regulation reforms in Vietnam and Asia in general. Vietnam disease-monitoring programs in livestock and domestic animals proved effective [63, 64] and provide a strong basis for further development. Expanding these programs into a national One Health surveillance strategy targeting high-risk wildlife value chain nodes and taxa would significantly enhance the country's capacity to detect and prevent zoonotic disease emergence.

To support this strategic shift and guide implementation, the following targeted recommendations are proposed for key stakeholder groups in Vietnam:

1. Policymakers:
  – Strengthen wildlife trade regulations
    • Implement stricter biosecurity measures along the wildlife value chain, such as mandatory health screening and quarantine protocols, stricter hygiene standards and waste disposal, and improved traceability
    • Update and enforce policies to reduce high-risk wildlife trade and farming and strengthen penalties for noncompliance
    • Promote legal and sustainable alternatives to wildlife consumption
  – Integrate One Health into national policy
    • Establish an effective cross-sectoral One Health surveillance framework linking public health, animal, and environmental sectors
    • Allocate funding for wildlife pathogen monitoring, outbreak preparedness, and disease prevention
    • Institutionalize data sharing mechanisms across ministries and agencies
    • Foster international collaboration to align with regional disease control efforts
  – Raise public awareness
    • Develop targeted campaigns to educate the public on zoonotic disease risks
    • Work with media and community leaders to reduce demand for wildlife products
2. Public health officials:
  – Expand surveillance and risk assessment
    • Incorporate wildlife monitoring into national disease surveillance programs and reporting systems
    • Conduct regular pathogen screening and collect quantitative data on pathogen exposure and impact for accurate risk assessments
    • Identify and prioritize high-risk nodes in the wildlife supply chain
  – Improve diagnostic and response capabilities
    • Establish rapid response protocols for field investigation and containment of emerging zoonotic outbreaks
    • Train health professionals in zoonotic disease recognition, biosafety practices, and outbreak investigation
    • Ensure adequate resources for early detection and outbreak containment
3. Researchers:
  – Advance pathogen detection methods
    • Develop unbiased metagenomic sequencing approaches to identify unknown pathogens
    • Improve diagnostic tools to detect zoonotic diseases in wildlife reservoirs
    • Improve laboratory infrastructure and capacity for high-containment research on high-risk pathogens
    • Conduct longitudinal studies on pathogen transmission along the wildlife value chain
  – Fill data gaps on zoonotic risks
    • Investigate environmental, socioeconomic, and behavioral drivers of wildlife-human disease spillover
    • Study host–pathogen interactions, including intermediate hosts and environmental reservoirs, to assess spillover risks
    • Provide evidence-based recommendations to guide risk management strategies

## Figures and Tables

**Table 1:** Number of bacteria, viruses, and other pathogens (protozoans and parasites) detected in each wildlife host family in Vietnam (data from our literature review) and globally (database from [23]).

Host order, family (common name)	Total number of bacteria (zoonotic)		Total number of viruses (zoonotic)		Number of other pathogens (zoonotic)	List of zoonotic pathogens reported in Vietnam
	Vietnam	Globally	Vietnam	Globally	Vietnam	
Amphibia (amphibians)						
Anura, **Bufonidae** (toads)	—	3 (3)	—	2 (0)	1 (0)	—
Anura, **Dicroglossidae** (fork-tongued frogs)	1 (1)	4 (4)	—	1 (0)	1 (0)	*Mycobacterium* sp.
Anura, **Ranidae** (true frogs)	—	3 (3)	—	6 (2)	1 (0)	—
Anura, **Rhacophoridae** (shrub frogs)	—	1 (1)	—	—	1 (0)	—
Caudata, **Salamandridae** (salamanders)	—	1 (1)	—	1 (0)	2 (0)	—
Aves (birds)						
Apodiformes, **Apodidae** (swifts)	—	2 (2)	1 (1)	—	—	**Influenza A virus**
Charadriiformes, **Scolopacidae** (sandpipers)	—	9 (9)	1 (1)	8 (4)	—	**Influenza A virus**
Columbiformes, **Columbidae** (pigeons and doves)	—	30 (21)	1 (1)	37 (17)	—	**Influenza A virus**
Galliformes, **Phasianidae** (jungle fowl, peacock, pheasant, quail)	—	48 (34)	3 (2)	50 (17)	—	**Influenza A virus** Avian orthoavulavirus 1
Passeriformes, **Dicruridae** (drongos)	—	—	1 (1)	—	—	**Influenza A virus**
Passeriformes, **Hirundinidae** (swallows)	—	4 (3)	1 (1)	10 (8)	—	**Influenza A virus**
Passeriformes, **Muscicapidae** (flycatchers)	—	5 (5)	1 (1)	7 (5)	—	**Influenza A virus**
Passeriformes, **Pellorneidae** (jungle babblers)	—	—	1 (1)	—	—	**Influenza A virus**
Passeriformes, **Pycnonotidae** (bulbuls)	—	1 (1)	1 (1)	3 (1)	—	**Influenza A virus**
Passeriformes, **Timaliidae** (babblers)	—	—	1 (1)	1 (1)	—	**Influenza A virus**
Pelecaniformes, **Ardeidae** (wading birds)	—	11 (11)	1 (1)	27 (13)	—	**Influenza A virus**
Struthioniformes, **Struthionidae** (ostriches)	1 (1)	18 (17)	1 (1)	12 (4)	1 (0)	**Influenza A virus** ***Clostridium perfringens***
Mammalia (mammals)						
Artiodactyla, **Cervidae** (deers)	—	60 (45)	—	48 (22)	—	—
Artiodactyla, **Giraffidae** (giraffes)	—	5 (4)	1 (0)	15 (7)	—	—
Artiodactyla, **Suidae** (pigs)	1 (1)	29 (27)	1 (0)	37 (18)	1 (1)	*Trichinella spiralis* *Ehrlichia* sp.
Carnivora, **Felidae** (cats)	—	33 (29)	5 (1)	29 (14)	3 (3)	*Paragonimus heterotremus* *Paragonimus skrjabini* *Paragonimus westermani* **Influenza A virus**
Carnivora, **Herpestidae** (mongooses)	—	6 (6)	—	7 (6)	1 (0)	—
Carnivora, **Mustelidae** (small carnivores)	—	44 (39)		26 (15)	1 (0)	—
Carnivora, **Ursidae** (bears)	7 (7)	28 (26)	1 (0)	23 (14)	—	*Enterococcus faecalis* ***Escherichia coli*** *Klebsiella pneumoniae* *Pseudomonas aeruginosa* *Acinetobacter* sp. *Staphylococcus* sp. *Streptococcus* sp.
Carnivora, **Viverridae** (civets)	—	3 (3)	3 (1)	10 (7)	1 (0)	**Influenza A virus**
Chiroptera, **Emballonuridae** (sheath-tailed bats)	—	—	3 (2)	8 (5)	—	European bat 1 lyssavirus **Lyssavirus rabies**
Chiroptera, **Hipposideridae** (leaf-nosed bats)	1 (1)	—	7 (4)	18 (11)	1 (0)	*Bartonella* sp. European bat 1 lyssavirus Hepatitis B related viruses Lyssavirus duvenhage **Lyssavirus rabies**
Chiroptera, **Megadermatidae** (false vampire bats)	1 (1)	—	—	2 (1)	—	*Bartonella* sp.
Chiroptera, **Miniopteridae** (bent-winged bats)	—	—	1 (1)	18 (7)	—	European bat 1 lyssavirus
Chiroptera, **Molossidae** (free-tailed bats)	—	1 (1)	5 (2)	21 (19)	—	European bat 1 lyssavirus **Lyssavirus rabies**
Chiroptera, Unidentified microbats	—	—	6 (0)	—	—	—
Chiroptera, **Pteropodidae** (fruit bats)	1 (1)	4 (4)	6 (3)	41 (27)	1 (0)	*Bartonella* sp. European bat 1 lyssavirus **Lyssavirus rabies** Nipah henipavirus
Chiroptera, **Rhinolophidae** (Horseshoe bats)	1 (1)	1 (1)	2 (2)	21 (14)	—	*Bartonella* sp. European bat 1 lyssavirus Lyssavirus duvenhage
Chiroptera, **Vespertilionidae** (microbats)	1 (1)	—	10 (3)	40 (22)	—	***Rickettsia* sp.** European bat 1 lyssavirus Lyssavirus duvenhage **Lyssavirus rabies**
Eulipotyphla, **Soricidae** (shrews)	1 (1)	17 (15)	2 (0)	16 (14)	1 (0)	***Escherichia coli***
Pholidota, **Manidae** (pangolins)	—	—	1 (0)	—	—	—
Primates, **Cercopithecidae** (Old World monkeys)	2 (2)	58 (52)	18 (13)	74 (50)	3 (3)	*Echinococcus ortleppi* ***Trichuris trichiura*** *Helicobacter heilmannii*-like *Helicobacter pylori* *Balantidium coli* Adenovirus Chikungunya virus Dengue virus Human gammaherpesvirus 4 **Japanese encephalitis virus** **Lyssavirus rabies** Macacine alphaherpesvirus 1 Mason-Pfizer monkey virus Measles morbillivirus Monkeypox virus **Paslahepevirus balayani** Primate T-lymphotropic virus 1 Simian foamy virus
Primates, **Hylobatidae** (gibbons)	—	5 (5)	1 (1)	11 (10)	—	Hepatitis B virus
Rodentia, **Hystricidae** (porcupines)	—	—	4 (0)	2 (2)	—	—
Rodentia, **Muridae** (rats and mice)	10 (10)	101 (67)	30 (7)	114 (68)	13 (7)	*Centrocestus formosanus* *Echinostoma cinetorchis* *Echinostoma hortense* *Echinostoma* sp. *Raillietina celebensis* *Trichinella spiralis* ***Escherichia coli*** ***Leptospira borgpetersenii*** ***Leptospira interrogans*** ***Leptospira* sp.** ***Orientia tsutsugamushi*** ***Rickettsia* sp. SFG** ***Rickettsia typhi*** *Bartonella* sp. ***Salmonella* sp.** *Trypanosoma lewisi* Aichivirus A Cowpox virus Duck-dominant Coronavirus Hantaan orthohantavirus Lymphocytic choriomeningitis mammarenavirus Seoul orthohantavirus Tick-borne encephalitis virus
Rodentia, **Sciuridae** (squirrels)	3 (3)	24 (20)	1 (0)	35 (24)	1 (0)	***Rickettsia* sp. SFG** *Bartonella* sp. ***Leptospira* sp.**
Rodentia, **Spalacidae** (bamboo rats)	—	—	10 (1)	—	—	**Influenza A virus**
Scandentia, **Tupaiidae** (treeshrews)	1 (1)	—	—	4 (0)	—	***Orientia tsutsugamushi***
Reptilia (reptiles)						
Crocodilia, **Crocodylidae** (crocodiles)	—	8 (7)	—	—	—	—
Squamata, **Agamidae** (Iguanian lizards)	—	10 (8)	—	2 (0)	2 (0)	—
Squamata, **Elapidae** (venomous snakes)	—	4 (3)	—	2 (1)	3 (0)	—
Squamata, **Gekkonidae** (common geckos)	2 (2)	1 (1)	—	1 (0)	1 (0)	***Escherichia coli*** ***Salmonella* sp.**
Squamata, **Colubridae** (snakes)	—	12 (12)	—	7 (3)	—	**—**
Squamata, **Pythonidae** (pythons)	34 (32)	12 (12)	—	5 (0)	—	*Acinetobacter baumanii* *Acinetobacter calcoaceticus* *Aeromonas hydrophila* *Chryseobacterium indologenes* *Citrobacter freundii* *Citrobacter koseri* *Corynebacterium jeikeium* *Enterobacter cloacae* *Enterococcus faecalis* ***Escherichia coli*** *Gemella haemolysans* *Klebsiella aerogenes* *Klebsiella oxytoca* *Klebsiella pneumoniae* *Kluyvera cryocrescens* *Kluyvera intermedia* *Kocuria rosea* *Morganella morganii* *Proteus mirabilis* *Proteus vulgaris* *Providencia rettgeri* *Pseudomonas aeruginosa* *Pseudomonas putida* *Staphylococcus lentus* *Staphylococcus xylosus* *Acinetobacter* sp. *Aeromonas* sp. *Bacillus* sp. *Enterobacter* sp. *Enterococcus* sp. *Pseudomonas* sp. *Staphylococcus* sp.
Squamata, **Xenopeltidae** (sunbeam snakes)	—	—	—	—	1 (0)	—

*Note:* The number of zoonotic pathogens for each type of pathogens is indicated in parentheses. Zoonotic pathogens reported in Vietnam are listed in the last column and Vietnam's priority zoonotic pathogens are highlighted in bold [21].

## Data Availability

The data that support the findings of this study are available in the supporting information of this article.

## Supporting Information

Supporting Information 1 Supporting Information S1: Pathogen inventory in Vietnam wildlife. Supporting Information S2: Zoonotic Disease Risk of Major Types of Wildlife Trade Chains & Points of Sale (adapted from Wikramanayake et al. (2021) [24]). Supporting Information S3: List of eligible records included in this study.
