# Supplementary material for: Wildlife Pathogens and Zoonotic Disease Risk Assessment in Vietnam: A Wildlife Trade Hotspot
Source: Transbound Emerg Dis. 2025 Jun 19;2025:4926262. doi: 10.1155/tbed/4926262 (PMC12202080; doi:10.1155/tbed/4926262)

**Supplementary Table 1**: Definitions of the 13 types of human-wildlife interfaces used in this study

| Interface | Definition |
| --- | --- |
| Wildlife farms | Facilities where captive wild animals are bred and raised for commercial purposes, i.e. with the intention of harvesting the animal or an animal product for commercial profit |
| Free-ranging animals in farm | Wild animals, including pests, ranging freely in farms breeding and raising livestock and domestic animals |
| Primate facilities | Facilities where large numbers of primates are bred, mostly for research purposes and international export |
| Restaurants | Places where wildlife meat is served to on-site consumers, either from live or dead wild animals sourced from the wild or from captive wildlife farms |
| Markets | Markets where wild animals are sold to consumers, either live or dead |
| Free-ranging animals in market | Wild animals, including pests, ranging freely in markets selling any type of goods to consumers |
| Wildlife rescue centers and sanctuaries | Facilities where wild animals, rescued from the illegal trade or from captive facilities, are rehabilitated, and sometimes released in the wild |
| Zoos and recreational parks | Facilities where wild animals are kept and presented to the public for recreational purposes |
| Bat guano farms and guano collection | Artificial bat roosts and natural caves where bat guano is collected to be sold as fertilizer |
| Free-ranging animals in natural habitat | Wild animals ranging freely in their natural habitat such as forests and crop fields |
| Free-ranging animals in human settlements | Wild animals ranging freely in human settlements, including villages, towns and cities |
| Confiscations from illegal trade | Wild animals confiscated from the illegal trade by law officers |
| International imports | Wild animals imported to Vietnam from neighboring countries for human consumption |

**Supplementary Table 2**: Class, order and common names of wildlife host species in which at least one pathogen/parasite was detected in Vietnam and number of publications with pathogen/parasite detection(s) in our literature review.

| Host class | Host order | Host common names | Number of publications with pathogen/parasite detection |
| --- | --- | --- | --- |
| Amphibia (amphibians) | Anura | Frogs and toads | 2 |
|  | Urodela | Salamanders | 1 |
| Aves (birds) | Apodiformes | Swifts, treeswifts and hummingbirds | 1 |
|  | Charadriiformes | Waders and shorebirds | 1 |
|  | Columbiformes | Pigeons and doves | 1 |
|  | Galliformes | Turkeys, chickens, quail, and other landfowl | 3 |
|  | Passeriformes | Passerines | 3 |
|  | Pelecaniformes | Aquatic birds | 1 |
|  | Struthioniformes | Ostriches | 3 |
| Mammalia (mammals) | Artiodactyla | Even-toed ungulates | 4 |
|  | Carnivora | Carnivores | 11 |
|  | Chiroptera | Bats | 19 |
|  | Eulipotyphla | Hedgehogs, moles and shrews | 4 |
|  | Pholidota | Pangolins | 1 |
|  | Primates | Lemurs, lorises, tarsiers, monkeys and apes | 13 |
|  | Rodentia | Gophers, mice, rats, squirrels, porcupines, beavers and chipmunks. | 33 |
|  | Scandentia | Treeshrews | 1 |
| Reptilia (reptiles) | Squamata | Lizards and snakes | 9 |

**Supplementary Table 3**: Number of pathogen/parasite species detected in each host class in Vietnam according to our literature review

| Host class | Type of pathogens | Number of pathogen species detected |
| --- | --- | --- |
| Amphibia (amphibians) | Parasites | 1 |
|  | Bacteria | 1 |
|  | Fungi | 2 |
|  | Protozoans | 0 |
|  | Viruses | 0 |
| Aves (birds) | Parasites | 0 |
|  | Bacteria | 1 |
|  | Fungi | 0 |
|  | Protozoans | 1 |
|  | Viruses | 3 |
| Mammalia (mammals) | Parasites | 16 |
|  | Bacteria | 18 |
|  | Fungi | 0 |
|  | Protozoans | 7 |
|  | Viruses | 81 |
| Reptilia (reptiles) | Parasites | 6 |
|  | Bacteria | 35 |
|  | Fungi | 0 |
|  | Protozoans | 0 |
|  | Viruses | 0 |

**Supplementary Table 4**: List and total number of pathogens detected and number of zoonotic pathogens per host families at each human-wildlife interface along the wildlife value chain in Vietnam. Zoonotic pathogens are in bold and priority zoonotic pathogens are in bold and underlined.

| **Interfaces** | Host families screened for pathogens | All pathogens detected | Total number of pathogens | Number of zoonotic pathogens |  |
| --- | --- | --- | --- | --- | --- |
| **Environment** | | | | | |
| **Free-ranging animals in natural habitat** | Salamandridae (salamanders) | *Batrachochytrium dendrobatidis*  *Batrachochytrium salamandrivorans* | 2 | 0 |  |
|  | Scolopacidae (sandpipers) | **Influenza A virus** | 1 | 1 |  |
|  | Columbidae (pigeons and doves) | **Influenza A virus** | 1 | 1 |  |
|  | Phasianidae (jungle fowl, peacock, pheasant, quail) | **Influenza A virus** | 1 | 1 |  |
|  | Dicruridae (drongos) | **Influenza A virus** | 1 | 1 |  |
|  | Muscicapidae (flycatchers) | **Influenza A virus** | 1 | 1 |  |
|  | Pellorneidae (jungle babblers) | **Influenza A virus** | 1 | 1 |  |
|  | Pycnonotidae (bulbuls) | **Influenza A virus** | 1 | 1 |  |
|  | Timaliidae (babblers) | **Influenza A virus** | 1 | 1 |  |
|  | Ardeidae (wading birds) | **Influenza A virus** | 1 | 1 |  |
|  | Suidae (pigs) | African Swine Fever virus  ***Trichinella spiralis*** | 2 | 1 |  |
|  | Felidae (cats) | ***Paragonimus heterotremus***  ***Paragonimus skrjabini***  ***Paragonimus westermani*** | 3 | 3 |  |
|  | Emballonuridae (sheath-tailed bats) | **European bat 1 lyssavirus**  Lyssavirus lagos  **Lyssavirus rabies** | 3 | 2 |  |
|  | Hipposideridae (leaf-nosed bats) | ***Bartonella* sp.**  Dakrong virus  **European bat 1 lyssavirus**  **Hepatitis B related viruses**  **Lyssavirus duvenhage**  **Lyssavirus rabies**  Unclassified bat cyclovirus  Xuan son mobatvirus | 8 | 5 |  |
|  | Megadermatidae (false vampire bats) | ***Bartonella* sp.** | 1 | 1 |  |
|  | Miniopteridae (bent-winged bats) | **European bat 1 lyssavirus** | 1 | 1 |  |
|  | Molossidae (free-tailed bats) | **European bat 1 lyssavirus**  Lyssavirus lagos  **Lyssavirus rabies** | 3 | 2 |  |
|  | Pteropodidae (fruit bats) | ***Bartonella* sp.**  Hemosporidians  **European bat 1 lyssavirus**  **Lyssavirus rabies** | 4 | 3 |  |
|  | Rhinolophidae (Horseshoe bats) | ***Bartonella* sp.**  **European bat 1 lyssavirus**  **Lyssavirus duvenhage** | 3 | 3 |  |
|  | Vespertilionidae (microbats) | **European bat 1 lyssavirus**  **Lyssavirus duvenhage**  **Lyssavirus rabies** | 3 | 3 |  |
|  | Soricidae (shrews) | *Trypanosoma sapaensis*  Cao Bang orthohantavirus | 2 | 0 |  |
|  | Muridae (rats and mice) | ***Trichinella spiralis***  ***Bartonella* sp.**  ***Leptospira* sp.**  ***Orientia tsutsugamushi*** | 4 | 4 |  |
|  | Sciuridae (squirrels) | *Hepatocystis* sp. | 1 | 0 |  |
|  | Agamidae (Iguanian lizards) | *Cosmocercoides tonkinensis*  *Falcaustra vietnamensis* | 2 | 0 |  |
|  | Xenopeltidae (sunbeam snakes) | *Macrobothriotaenia ficta* | 1 | 0 |  |
| **Free-ranging animals in farms** | Soricidae (shrews) | ***Escherichia coli*** | 1 | 1 |  |
|  | Muridae (rats and mice) | ***Bartonella* sp.**  ***Escherichia coli***  ***Salmonella* sp.**  Scotophilus bat coronavirus 512 | 4 | 3 |  |
|  | Gekkonidae (common geckos) | ***Escherichia coli***  ***Salmonella* sp.** | 2 | 2 |  |
| **Free-ranging animals in markets** | Muridae (rats and mice) | ***Escherichia coli***  ***Leptospira interrogans***  ***Orientia tsutsugamushi***  ***Trypanosoma lewisi***  *Rocahepevirus ratti* | 5 | 4 |  |
| **Free-ranging animals in human settlements** | Hirundinidae (swallows) | **Influenza A virus** | 1 | 1 |  |
|  | Hipposideridae (leaf-nosed bats) | Hemosporidians | 1 | 0 |  |
|  | Pteropodidae (fruit bats) | PREDICT_CoV-17  PREDICT_CoV-35  *Pteropus lylei*-associated alphaherpesvirus | 3 | 0 |  |
|  | Muridae (rats and mice) | ***Bartonella* sp.**  ***Escherichia coli***  ***Leptospira interrogans***  ***Leptospira* sp.**  ***Orientia tsutsugamushi***  ***Rickettsia* sp. SFG**  ***Rickettsia typhi***  ***Trypanosoma lewisi***  *Rocahepevirus ratti*  **Seoul orthohantavirus**  Unclassified hantavirus | 11 | 9 |  |
|  | Sciuridae (squirrels) | ***Bartonella* sp.**  ***Leptospira* sp.**  ***Rickettsia* sp. SFG** | 3 | 3 |  |
| **Wildlife sources** | | | | | |
| **Wildlife farms** | Bufonidae (toads) | *Pseudoacanthocephalus nguyenthileae* | 1 | 0 |  |
|  | Dicroglossidae (fork-tongued frogs) | *Pseudoacanthocephalus nguyenthileae*  ***Mycobacterium* sp.** | 2 | 1 |  |
|  | Ranidae (true frogs) | *Pseudoacanthocephalus nguyenthileae* | 1 | 0 |  |
|  | Rhacophoridae (shrub frogs) | *Pseudoacanthocephalus nguyenthileae* | 1 | 0 |  |
|  | Struthionidae (ostriches) | ***Clostridium perfringens***  *Cryptosporidium avium*  **Influenza A virus** | 3 | 2 |  |
|  | Viverridae (civets) | Unclassified kobuvirus | 1 | 0 |  |
|  | Cercopithecidae (Old World monkeys) | PREDICT_RbdV-15 | 1 | 0 |  |
|  | Hystricidae (porcupines) | Avian coronavirus  Murine coronavirus  PREDICT_RbdV-15  Scotophilus bat coronavirus 512 | 4 | 0 |  |
|  | Spalacidae (bamboo rats) | Avian coronavirus  Longquan Aa mouse coronavirus  Murine coronavirus  Scotophilus bat coronavirus 512  Unclassified bamboo rat kobuvirus  Unclassified hepacivirus | 6 | 0 |  |
|  | Elapidae (venomous snakes) | *Porrorchis houdemeri*  *Pseudoacanthocephalus nguyenthileae*  *Sphaerechinorhynchus maximesospinus* | 3 | 0 |  |
|  | Gekkonidae (common geckos) | *Pseudoacanthocephalus nguyenthileae* | 1 | 0 |  |
|  | Pythonidae (pythons) | ***Acinetobacter baumanii***  ***Acinetobacter calcoaceticus***  ***Acinetobacter* sp.**  ***Aeromonas hydrophila***  ***Aeromonas* sp.**  ***Bacillus* sp.**  *Brevibacillus brevis*  ***Chryseobacterium indologenes***  ***Citrobacter freundii***  ***Citrobacter koseri***  ***Corynebacterium jeikeium***  ***Enterobacter cloacae***  ***Enterobacter* sp.**  ***Enterococcus faecalis***  ***Enterococcus* sp.**  ***Escherichia coli***  ***Gemella haemolysans***  ***Klebsiella aerogenes***  ***Klebsiella oxytoca***  ***Klebsiella pneumoniae***  ***Kluyvera cryocrescens***  ***Kluyvera intermedia***  ***Kocuria rosea***  ***Morganella morganii***  ***Proteus mirabilis***  ***Proteus vulgaris***  ***Providencia rettgeri***  ***Pseudomonas aeruginosa***  ***Pseudomonas putida***  ***Pseudomonas* sp.**  *Shewanella putrefaciens*  ***Staphylococcus lentus***  ***Staphylococcus* sp.**  ***Staphylococcus xylosus*** | 34 | 32 |  |
| **Primate facilities** | Cercopithecidae (Old World monkeys) | ***Trichuris trichiura***  ***Helicobacter heilmannii*-like**  ***Helicobacter pylori***  ***Balantidium coli***  **Adenovirus**  Cercopithecine alphaherpesvirus 9  **Chikungunya virus**  **Dengue virus**  **Human gammaherpesvirus 4**  **Japanese encephalitis virus**  **Lyssavirus rabies**  **Macacine alphaherpesvirus 1**  Macacine betaherpesvirus 3  **Mason-Pfizer monkey virus**  **Measles morbillivirus**  **Monkeypox virus**  **Paslahepevirus balayani**  **Primate T-lymphotropic virus 1**  Rocahepevirus ratti  **Simian foamy virus**  Unclassified rotavirus | 21 | 18 |  |
| **Bat guano farm and guano collection** | Molossidae (free-tailed bats) | PREDICT_CoV-47  PREDICT_CoV-82 | 2 | 0 |  |
|  | Unidentified Chiroptera | PREDICT_CoV-17  PREDICT_CoV-35  PREDICT_CoV-47  PREDICT_CoV-99  PREDICT_PMV-63  Scotophilus bat coronavirus 512 | 6 | 0 |  |
|  | Vespertilionidae (microbats) | PREDICT_PMV-13  PREDICT_PMV-66  Scotophilus bat coronavirus 512  Unclassified bat kobuvirus  Unclassified Kobuvirus  Unclassified Parechovirus  Unclassified posa-like virus | 7 | 0 |  |
| **Wildlife distribution and sales** | | | | | |
| **Confiscation from illegal trade** | Ursidae (bears) | PREDICT_HV-20 | 1 | 0 |  |
|  | Manidae (pangolins) | SARS-CoV-2 related Coronavirus | 1 | 0 |  |
| **Markets** | Apodidae (swifts) | **Influenza A virus** | 1 | 1 |  |
|  | Phasianidae (jungle fowl, peacock, pheasant, quail) | **Influenza A virus** | 1 | 1 |  |
|  | Muridae (rats and mice) | ***Bartonella* sp.**  ***Leptospira* sp.**  **Aichivirus A**  Beilong jeilongvirus  China Rattus coronavirus HKU24  **Duck-dominant Coronavirus**  Longquan Aa mouse coronavirus  Murine coronavirus  Murine respirovirus  Rodent coronavirus  Scotophilus bat coronavirus 512  **Seoul orthohantavirus**  Unclassified betacoronavirus  Unclassified Cardiovirus  Unclassified Hunnivirus  Unclassified Mosavirus  Unclassified Mupivirus  Unclassified Parabovirus  Unclassified Parechovirus  Unclassified Rabovirus  Unclassified Rosavirus | 21 | 5 |  |
| **Mixed interfaces:**  **Markets OR Free-ranging animal in natural habitat** | Muridae (rats and mice) | **Cowpox virus**  **Lymphocytic choriomeningitis** **mammarenavirus**  Rocahepevirus ratti  **Tick-borne encephalitis virus**  Unclassified betacoronavirus  Unclassified hantavirus  Unclassified hepacivirus  Unclassified Kobuvirus  Unclassified posa-like virus | 9 | 3 |  |
| **International import** | Muridae (rats and mice) | ***Leptospira borgpetersenii***  ***Leptospira interrogans*** | 2 | 2 |  |
| **Wildlife consumption** | | | | | |
| **Restaurants** | Phasianidae | Avian coronavirus  **Avian orthoavulavirus 1** | 2 | 1 |  |
|  | Viverridae | PREDICT_HV-17 | 1 | 0 |  |
|  | Muridae (rats and mice) | China Rattus coronavirus HKU24  Longquan Aa mouse coronavirus  Murine coronavirus  PREDICT_PMV-58  PREDICT_RbdV-19 | 5 | 0 |  |
|  | Spalacidae | **Influenza A virus**  PREDICT_RbdV-16  PREDICT_RbdV-17  PREDICT_RbdV-18 | 4 | 1 |  |
| **Wildlife conservation and recreation** | | | | | |
| **Zoos and recreational parks** | Giraffidae | Lumpy skin disease virus | 1 | 0 |  |
|  | Felidae | Felid alphaherpesvirus 1  Feline calicivirus  Feline foamy virus  Feline parvovirus  **Influenza A virus** | 5 | 1 |  |
|  | Sciuridae | Murine coronavirus | 1 | 0 |  |
| **Wildlife rescue centers and sanctuaries** | Ursidae | **Acinetobacter sp.**  **Enterococcus faecalis**  **Escherichia coli**  **Klebsiella pneumoniae**  **Pseudomonas aeruginosa**  **Staphylococcus sp.**  **Streptococcus sp.** | 7 | 7 |  |
|  | Viverridae | **Influenza A virus** | 1 | 1 |  |
|  | Cercopithecidae (Old World monkeys) | **Echinococcus ortleppi** | 1 | 1 |  |
|  | Hylobatidae (gibbons) | **Hepatitis B virus** | 1 | 1 |  |

**Supplementary Table 5**: Evidences of viral cross-taxa transmission at different human-wildlife interfaces in Vietnam

| **Pathogen** | **Natural host** | **New host** | **Interface** | **Source** |
| --- | --- | --- | --- | --- |
| PREDICT_RbdV-15 | Cercopithecidae (Old World monkeys) | Hystricidae (porcupines) | Wildlife farms | (Wildlife Conservation Society 2020) |
| Avian coronavirus | Birds | Hystricidae (porcupines) | Wildlife farms | (Huong Nguyen Quynh et al. 2020a) |
| Avian coronavirus | Birds | Spalacidae (bamboo rats) | Wildlife farms | (Huong Nguyen Quynh et al. 2020a) |
| Scotophilus bat coronavirus 512 | Bats | Hystricidae (porcupines) | Wildlife farms | (Huong Nguyen Quynh et al. 2020a) |
| Scotophilus bat coronavirus 512 | Bats | Spalacidae (bamboo rats) | Wildlife farms | (Huong Nguyen Quynh et al. 2020a) |
| Scotophilus bat coronavirus 512 | Bats | Muridae (rats and mice) | Free-ranging animal in farms,  Markets | (Huong Nguyen Quynh et al. 2020a) |
| Duck-dominant coronavirus | Birds | Muridae (rats and mice) | Markets | (Wildlife Conservation Society 2020) |

**Supplementary Figure 1**: Risk ranking of traded taxa based on the number of zoonotic pathogens and the number of priority zoonotic pathogens reported globally in each wildlife family (“zoonotic risk”, High risk = ≥50 known pathogens and ≥40 zoonotic pathogens; Medium risk = 20-49 known pathogens and/or 15-39 zoonotic pathogens; Low risk = ≤19 known pathogens and ≤14 zoonotic pathogens; “priority zoonotic risk”, High risk = ≥5 priority zoonotic pathogens; Medium risk = 4-2 priority zoonotic pathogens; Low risk = 1 or no priority zoonotic pathogen). We selected the highest risk category between the zoonotic risk and the priority zoonotic risk as the final “traded taxa risk” for each family.


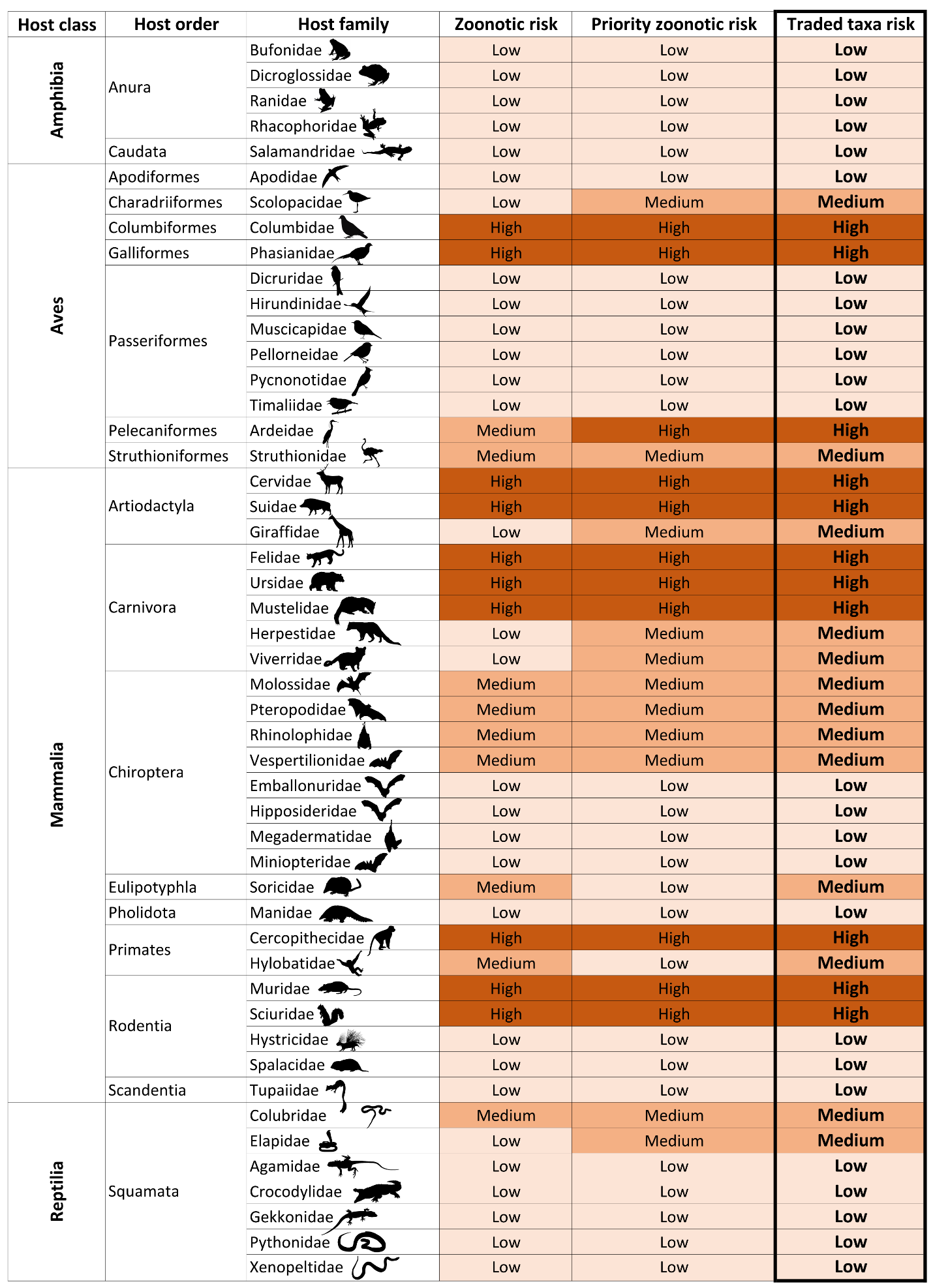

Supplement: Supporting Information 2 — Supporting Information Tables S1–S5 and Figure S1. [file 4926262.f2.docx]
